# Can Secondary Adhesive Capsulitis Complicate Calcific Tendinitis of the Rotator Cuff? An Ultrasound Imaging Analysis

**DOI:** 10.3390/clinpract14020045

**Published:** 2024-03-28

**Authors:** Giovanni Tuè, Oriana Masuzzo, Francesco Tucci, Marco Cavallo, Anna Parmeggiani, Fabio Vita, Alberto Patti, Danilo Donati, Alessandro Marinelli, Marco Miceli, Paolo Spinnato

**Affiliations:** 1 Diagnostic and Interventional Radiology, IRCCS Istituto Ortopedico Rizzoli, 40136 Bologna, Italy; 2 Shoulder and Elbow Unit, IRCCS Istituto Ortopedico Rizzoli, 40136 Bologna, Italy; 3 1st Orthopaedic and Traumatologic Clinic, IRCCS Istituto Ortopedico Rizzoli, Via Giulio Cesare Pupilli 1, 40136 Bologna, Italy; 4 Physical Therapy and Rehabilitation Unit, Policlinico di Modena, 41125 Modena, Italy

**Keywords:** adhesive capsulitis, diagnostic imaging, diagnostic ultrasound, shoulder, shoulder pain, tendinopathy

## Abstract

Background: Adhesive capsulitis (AC) of the glenohumeral joint is a recognized cause of pain associated with both active and passive restricted ranges of movement. AC can be subdivided into primary and secondary forms. Trauma, surgery, immobilization, and diabetes mellitus are the leading well-recognized causes of secondary AC. Calcific tendinitis/tendinitis (CT) of the rotator cuff is considered a possible trigger for AC, as reported in a few previous articles. However, there are no original investigations that assess the frequency and characteristics of this association. The aim of our research was to evaluate the presence of AC in a cohort of patients with a known CT condition of the rotator cuff by an ultrasound (US) examination. Materials and methods: We prospectively enrolled all the patients admitted at our single institution (October 2022–June 2023) for the preoperative US evaluation of a known CT condition. In these patients, we searched for parameters related to secondary AC. An axillary pouch (AP) thickness equal to or greater than 4 mm (or greater than 60% of the contralateral AP) was considered diagnostic of AC. Moreover, rotator interval (RI) thickness and the presence of effusion within the long-head biceps tendon (LHBT) sheath was also assessed in all patients. Results: A total of 78 patients (54F, 24M—mean age = 50.0 and range = 31–71 y.o.) were enrolled in the study. In 26 of those patients (26/78—33.3%), US signs of AC were detected. Notably, the mean AP thickness in patients with AC and CT was 3.96 ± 1.37 mm (Group 1) and 2.08 ± 0.40 mm in patients with CT only (Group 2). RI thickness was significantly greater in patients with superimposed AC: 2.54 ± 0.38 mm in Group 1 and 1.81 ± 0.41 mm in Group 2 (*p* < 0.00001). Moreover, effusion within the LHBT was significantly more frequently detected in patients with AC: 84.61% in Group 1 versus 15.79% in Group 2—*p* < 0.00001. Conclusion: US signs of AC are found in one-third of patients with CT of the rotator cuff, demonstrating that AC represents a frequent complication that should be routinely evaluated during US investigation to provide more personalized treatment strategies.

## 1. Introduction

One of the most common causes of non-traumatic shoulder pain is calcific tendinitis (CT). The disease is characterized by elemental calcium phosphate crystals, predominantly hydroxyapatite. Overall, 10 to 42% of the shoulders of patients with chronic pain had a CT. Calcific deposits can also go undetected in 3% of the shoulders of patients without pain. Women are more frequently afflicted than males by CT, and patients are typically between the ages of 30 and 60. There is frequent bilateral interaction, and CT is unrelated to manual labor or trauma [1].

This relatively common disorder typically affects the rotator cuff tendons or, less frequently, other tendons in other skeletal sites [2,3,4,5,6,7,8,9,10,11]. Moreover, when the disease affects a peri-articular structure such as capsular fibers or ligaments, it is commonly called calcific periarthritis [12]. The supraspinatus tendon is the most frequently affected rotator cuff tendon, followed by the infraspinatus and subscapularis tendons. Although the size and presence of bursitis on imaging have been demonstrated to be connected with the pain phases, all the factors contributing to the onset of symptoms remain partially unclear.

The diagnosis is usually clinically suspected, and imaging plays a role in confirming the diagnosis. Regardless of the degree of chronicity, conventional radiography is typically the first imaging technique to assess patients who report joint pain and limited range of motion. Whenever necessary, customized views are taken based on the joint, and two orthogonal views are often obtained for radiographs [4]. Compared to Magnetic Resonance Imaging (MRI) and standard radiography, ultrasonography is the most effective way to assess all phases of calcific tendinitis. Depending on the calcification stage, ultrasonography displays calcific deposits as foci with a hyperechogenic rim, interior hypo- or hyperechogenicity, and posterior acoustic shadowing [4].

Numerous treatments have been suggested over the years because the condition can cause excruciating agony. Today, ultrasound-guided irrigation is regarded as the gold standard treatment for CT of the rotator cuff.

One of the possible complications of CT is thought to be adhesive capsulitis (AC) [13], commonly known as “frozen shoulder”. AC is an invalidating disease characterized by an inflammation of the glenohumeral joint with fibroblastic reaction and scar tissue formation [14]. Clinically, this results in progressive pain and reduced range of motion in both active and passive movements. The prevalence in the general population is 2–5% and tends to be more common in women of peri-menopausal age (peak of incidence 40–60 years old) [14].

The disease recognizes three different and subsequent phases: a freezing phase (stage I; average duration of 3–9 months) characterized by severe pain worst at night with progressive stiffness and initial reduction in range of movements (ROM); a frozen phase (stage II; average duration of 9–18 months) characterized by peak of shoulder stiffness with complete loss of active and passive ROM; a thawing phase (stage III) with progressive resolution of shoulder stiffness and restoration of ROM [14].

AC can be classified as primary (or idiopathic) and secondary forms. In particular, risk factors for secondary AC are represented by previous trauma or surgery of the shoulder, diabetes, hypothyroidism, hypoadrenalism, hyperparathyroidism, and other hormone imbalances [15].

The recognition of AC as a possible complication of CT is still very limited. Indeed, only a few previous authors recognize this possible association in review articles or treatment-focused papers [13,16,17]. Regardless, to date, no original research has been focused on analyzing the association between CT and AC to determine the prevalence and characteristics of this possible complication.

The ultrasound (US) of the shoulder is considered to be the imaging method of choice for the assessment of CT of the rotator cuff tendons. Moreover, recent research has revealed that the US examination is an effective and reliable tool in diagnosing AC of the glenohumeral joint, with several signs of disease recognized [18,19,20,21].

The goal of our research was to systematically check the US signs of AC in patients with known CT of the rotator cuff to assess the frequency and characteristics of this complication.

## 2. Materials and Methods

We prospectively enrolled all the consecutive patients submitted to pre-operative US for the ultrasound-guided treatment (irrigation) of CT at our single Institution in the period ranging from October 2022 to June 2023. Patients with pain in the contralateral shoulder have been excluded because of the necessity of a contralateral non-affected side comparison.

### 2.1. Ultrasound Equipment

All the procedures have been performed with an Ultrasound Logiq E10 General Electric Healthcare (GE HealthCare, Chicago, IL, USA) equipment, with a 6–15 Mhz linear probe using a pre-imposed musculoskeletal setting calibrated for the shoulder.

### 2.2. Ultrasound Analysis

We evaluated the following ultrasound features:

- The presence of calcific deposit and its location;
- Calcific deposit maturation stage, according to the classification proposed by Chiou et al. [21]: stage I (arc-shaped with complete posterior acoustic shadowing), stage II (fragmented or punctate with partial posterior acoustic shadowing), stage III (nodular, without posterior shadowing) and stage IV (echogenic with cystic degenerative areas and without posterior shadowing);
- Axillary pouch (AP) maximum thickness: evaluated in the supine or sitting position with the probe placed longitudinally on the mid-axillary line along the neck of the humerus;
- Rotator interval (RI) maximum thickness: evaluated in the sitting position with the arm in a neutral position, elbow flexed, and hand palm on the knee. RI thickness was evaluated in a scan that included LHBT, supraspinatus, and subscapularis tendons by measuring the distance between LHBT outer contour and peri bursal fat;
- Effusion within the LHBT sheath: evaluated in the same position of the RI, with the probe placed in the axial plane in the bicipital groove.

The AP thickness of the affected size equal to or greater than 4 mm and less than 4 mm but greater than 60% of the contralateral non-affected shoulder was considered diagnostic of AC [14,15].

Based on this parameter, we also evaluated the other recognized US signs of AC, both RI thickness and effusion within the LHBT sheath in the group of patients with CT and superimposed AC (Group 1) and in the group of patients with CT alone (Group 2). This is to reinforce the diagnosis of AC and to confirm these US signs as a supportive feature (in addition to AP thickening) contributing to this diagnosis.

All the US examinations were performed by an expert musculoskeletal radiologist with 15 years of experience in the field (PS), helped by two senior radiology residents (GT, OM).

### 2.3. Statistical Analysis

Descriptive vital statistics were used to characterize the demographic distribution of the included population.

The Mann–Whitney U test was used to compare AP thickness and RI thickness, while Fisher’s exact test was used to compare the presence/absence of effusion within the LHBT sheath among the two groups (CT with secondary AC vs. CT without secondary AC).

A *p*-value < 0.05 was considered to indicate statistically significant differences. All tests were two-tailed.

### 2.4. Clinical Assessment

Among all the patients, two orthopedic surgeons specialized in shoulder and elbow pathologies (AM, MC) clinically evaluated the patients at the moment of hospital admission.

### 2.5. Ethics

Our institution’s local IRB (IRCCS Istituto Ortopedico Rizzoli, Bologna, Italy) approved this prospective study (code 379/2022/Sper/IOR) in August 2022. Each patient signed informed consent at the time of the hospital admission.

## 3. Results

### 3.1. Patients Demographics

A total of 78 patients with painful CT located within rotator cuff tendons have been included in our study: 54 females (69.2%) and 24 males (30.8%), with a mean age of 50.0 years old (±9.08 standard deviation—range 31–71).

### 3.2. Calcific Deposit Maturation Stage

CT was located in 59 patients in the supraspinatus (75.64%), in 7 patients in the subscapularis (8.97%), in 3 patients in the infraspinatus (3.85%), in 6 patients in both the supraspinatus and infraspinatus tendons (7.69%), and in 3 patients in both the supraspinatus and subscapularis tendons (3.85%).

**Figure 1:**
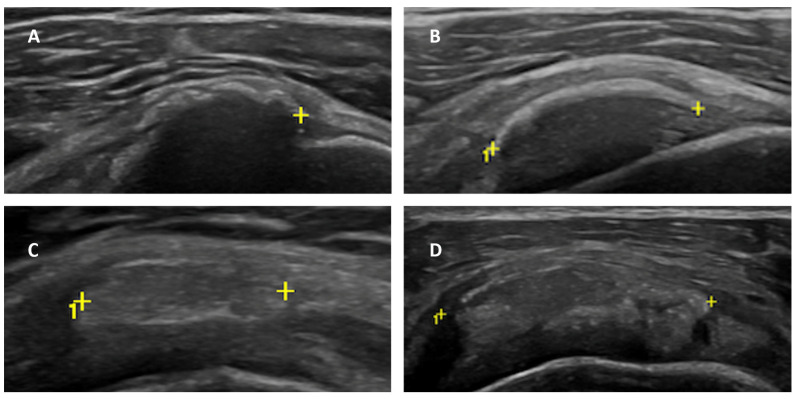
Calcific maturation stage: stage 1 (**A**)—arc-shaped with strong posterior shadowing); Stage 2 (**B**)—fragmented or punctate with partial posterior shadowing); Stage 3 (**C**)—nodular without posterior shadowing); Stage 4 (**D**)—nodular with cystic degenerative areas and without posterior shadowing). Symbol (+) represents measurement landmarks in ultrasound images.

Regarding the maturation stage of CT, stage I was found in 5 patients (6.41%), stage II in 21 patients (26.92%), stage III in 38 patients (48.71%), and stage IV in 14 patients (17.94%)—Figure 1, Table 1.

### 3.3. Axillary Pouch (AP) Thickness

**Figure 2:**
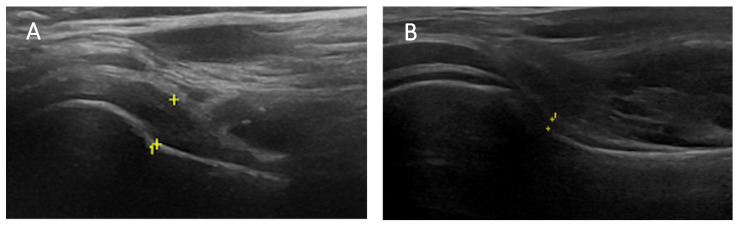
AP thickness is evaluated by positioning the probe longitudinally along the mid-axillary line, measuring the distance between the cortical line of the humeral neck to the outer margin of the glenohumeral joint. On the left (**A**) is a thickened AP (5.1 mm) of the affected shoulder. On the right (**B**) is a normal AP (1.1 mm) of the contralateral shoulder. Symbol (+) represents measurement landmarks in ultrasound images.

Among the 78 patients, 26 (26/78—33.33%) presented a thickened AP: 11 with an AP thickness >4 mm (11/26—42.3%), and 15 with an AP thickness <4 mm and >60% of the contralateral non-affected shoulder (15/26—57.7%). These patients received an ultrasound diagnosis of secondary AC and were collocated in group 1 of the study. The mean AP thickness observed in group 1 was 3.96 ± 1.37 mm (range 2.0–8.0 mm)—Figure 2.

The other 52 patients presented an AP thickness of less than 4 mm and less than 60% compared to the contralateral non-affected shoulder. In these patients enrolled in group 2 of the study without adhesive capsulitis, the mean AP thickness value observed was 2.08 ± 0.40 mm.

The Mann–Whitney U test revealed a normal distribution (U-value: 14.5) with significant differences among the AP thicknesses of the two groups (*p*-value < 0.00001).

### 3.4. Rotator Interval (RI) Thickness and Effusion within the LHBT Sheath

In both groups, RI and effusion within LHBT were then separately evaluated and statistically compared as follows.

**Figure 3:**
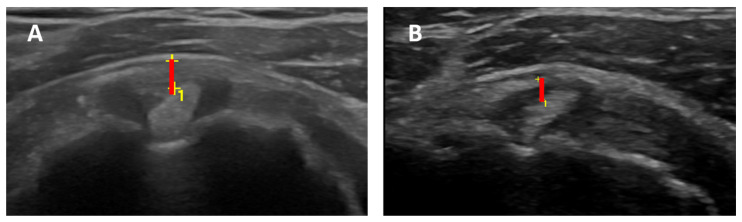
RI thickness (red lines with + respresents landmarks measurements) evaluated by positioning the transducer to visualize the LHBT between the supraspinatus and the subscapularis tendons, measuring the distance among the outer margin of LHBT and peribursal fat. On the left (**A**) is a thickened RI (1.6 mm) of the affected shoulder. On the right (**B**) is a normal RI (0.7 mm) of the contralateral shoulder.

In group 1, the RI thickness was significantly greater than in group 2 (2.54 ± 0.38 mm vs. 1.81 ± 0.41 mm; U-value 52—*p*-value < 0.00001)—Figure 3.

**Figure 4:**
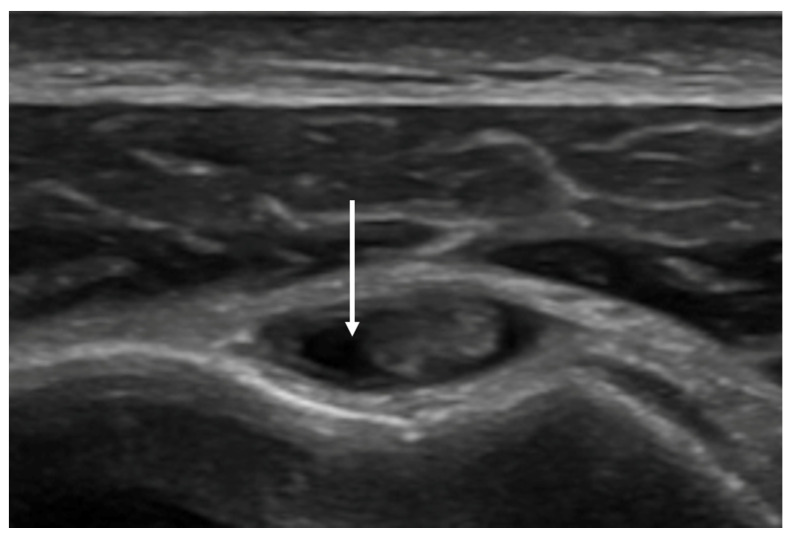
Effusion within the LHBT sheath (arrow). The probe is positioned in the short-axis view, and the bicipital groove is located between the greater (GT) and lesser (LT) humeral tuberosities.

Evidence of effusion within the LHBT sheath, compared among the two groups through Fisher’s exact test, was significantly more frequent in group 1 than in group 2 (84.61% vs. 15.79%; *p*-value < 0.00001)—Figure 4.

The main US findings related to AC are reported in Table 2.

### 3.5. Clinical Notes

In all the patients (78/78), a significant reduction in range of motion was found by the orthopedic surgeons because of intense pain, making a complete clinical evaluation of passive range of motion (clinical feature typical of AC) challenging to establish.

**Figure 5:**
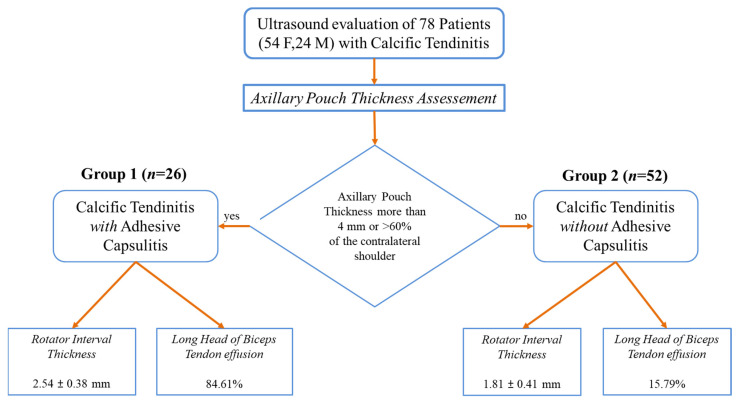
Schematic representation of the demographics and categorization into group 1 (CT with AC) and group 2 (CT without AC) according to AP thickness and the relative RI thickness and LHBT effusion percentage in the two groups.

In Figure 5, we provide a visual schematized summary of the current study.

## 4. Discussion

To the best of our knowledge, this is the first original research focused on assessing the diagnosis of secondary AC in patients with CT of the rotator cuff. Indeed, the association between CT of the rotator cuff and glenohumeral AC is reported even in recent review articles as a possible and not wholly understood association [22].

Our study included 78 consecutive patients who presented at our institution for shoulder pain related to CT with the purpose of ultrasound-guided irrigation planning. The results of the current research revealed that AC may complicate the condition of rotator cuff CT in about one-third of patients. In our cohort, patients with AC secondary to CT presented more frequently advanced maturation of the CT stages (38.5% in stage III, 34.6% in stage IV, according to Chiou et al.) [20].

MRI is considered the gold standard imaging tool for assessing or confirming the condition of glenohumeral AC [23]. Nonetheless, several recent studies have suggested the emerging role of diagnostic ultrasound in detecting AC [19]. The availability of ultrasound, its lower costs, and its increasing availability render it a very promising tool to assess both CT (as already known) and also AC. Still, in usual clinical practice, it is uncommon for US practitioners to evaluate the parameters that have been demonstrated to be related to an AC diagnosis, mainly if a diagnosis of rotator cuff CT is already obtained.

The results of this analysis suggest physicians involved in the shoulder US examinations search for AC signs in patients with rotator cuff CT. Indeed, this additional diagnosis/complication may be relevant to address the subsequent treatment strategies correctly. Physical therapy can be adjusted and planned to improve the AC condition rather than for CT alone. Moreover, a proper ultrasound-guided treatment should be proposed for these patients (e.g., hydrodistension). Notably, among the previously well-known ultrasound-guided treatments for a painful shoulder, a combined ultrasound-guided interventional procedure for both CT and AC treatment has recently been described [15].

While according to the guidelines of the European Society of Musculoskeletal Radiology, a radiological diagnosis of AC can be made only with MRI [22], several studies have recently demonstrated the diagnostic reliability through correct US exams, evaluating the three parameters strongly correlated with AC: increased AP thickness, increased RI thickness, and effusion within the LHBT sheath [19,20]. According to the existing literature, AP thickness is the most strongly associated US parameter among those mentioned above [21,22].

Our results inherent to AP thickness, RI thickness, and frequency of LHBT effusion in patients with AC almost overlap with those reported in the literature [21]. This reinforces the value of these two secondary US signs as a marker of AC in addition to AP thickness.

Because of this, we think that a US evaluation of these parameters should become part of regular ultrasound diagnostic exams in patients with shoulder pain and reduced range of motion related to CT to exclude the presence of secondary AC, considering that in one-third of the patients enrolled in our study for CT, we diagnosed secondary AC.

This study has some limitations. First of all, ultrasound is strongly operator-dependent so that measurements can differ among practitioners. However, all three main parameters were evaluated by a practitioner experienced in musculoskeletal radiology. Furthermore, a correct clinical evaluation of the potential reduction in passive range of motion (a clinical distinctive feature of adhesive capsulitis) in these patients was not feasible because of severe pain at the presentation. A more extensive observational study is needed to validate the results reported in our study. Moreover, our study is focused only on the diagnostic aspect of these pathologies, and we did not evaluate the results of ultrasound-guided treatments performed after the ultrasound diagnosis. This could be a valid aim for future research, particularly to know if the combined ultrasound-guided treatment of AC and CT leads to a better outcome compared to the treatment of CT alone. Lastly, even if we firmly suspect that CT was the cause of secondary AC in all the included patients, we cannot be sure that in some of them, other interfering causes may have played a role in AC development. 

The rationale of this association (AC + CT) can be supposed, but it is still not completely understood. The probable causes of AC superimposition in patients affected by CT of the rotator cuff could be related to the local inflammatory processes that may involve the glenohumeral joint capsule, as well as the immobilization or reduction in movements in these patients because of pain [16]. Moreover, some hormonal imbalances (such as hypothyroidism) are thought to be a risk factor for both conditions (AC and CT) and may be considered as a possible triggering factor [24]. The physical features of the calcium deposit will significantly influence the patient’s type of discomfort and may also influence the secondary AC superimposition. Indeed, if the calcium deposit quickly turns into a liquid form, the process will usually be fast. If the calcium deposit is dry and firm, a chronic variant, with less severe discomfort and a restricted range of motion in the shoulder, will be created, which can even last for several months. In the latter case, a secondary frozen shoulder (AC) would be more likely to develop.

Evaluating these three parameters is, according to us, of primary importance in clinical practice to avoid a missed diagnosis of secondary AC in patients with CT to propose the best treatment to the patient [17].

We believe that the diagnosis of AC, in addition to a known condition of CT, may greatly impact patients’ management. First of all, in patients with AC, a more intensive rehabilitation program should be proposed compared with patients with CT alone: therapeutic exercise, joint mobilization, scapulothoracic girdle and rotator cuff exercises, and stretching [25]. Importantly, in recent years, several ultrasound-guided treatments have been demonstrated to be safe and effective in the treatment of shoulder AC (e.g., hydrodistention, drug injection, platelet-rich plasma-based treatments) that can be performed in combination with physical therapies [26,27]. Since ultrasound-guided treatments are proven to be effective for treating CT and AC, the recognition of these conditions simultaneously in US studies may allow a direct ultrasound-guided combined treatment and/or proper subsequent specific rehabilitation programs [16,28,29,30,31,32,33]. Spinnato et al., in 2023, described a new ultrasound-guided technique to safely and effectively treat these two conditions in a single session, named the ‘Rizzoli’ technique [16]. However, a more extensive series is needed, particularly when comparing patients treated with CT irrigation alone and patients treated with irrigation plus hydrodistention (‘Rizzoli’ technique). In the past decades, when ultrasound-guided treatments were not yet validated (especially for AC), these combined treatments have been already proposed by Chen et al. using arthroscopic debridement of the glenohumeral joint capsule and performing multiple punctures into the CT [18].

## 5. Conclusions

This is the first original research focused on the assessment of AC in patients with CT of the rotator cuff. The results of our ultrasound imaging analysis confirm that AC can complicate CT in a relevant number of patients. The knowledge of this frequent association should be considered by clinicians (orthopedics, radiologists, and physiatrists) involved in the care of these patients. Ultrasound is a valid tool for the diagnosis of both conditions (AC and CT), and it can be evaluated in the same session. This is with the goal of a personalized and more effective treatment strategy for these patients, considering the possibility of specific mini-invasive ultrasound-guided treatments and rehabilitations for the two clinical conditions.

## Figures and Tables

**Table 1:** Calcific deposition stage frequencies, according to Chiou et al. [21], in patients with CT + AC (Group 1) and CT alone (Group 2).

Calcific Deposition Stage	Group 1 CT + AC (*n* = 26)	Group 2 CT (*n* = 52)
Stage I	2 (7.69%)	3 (5.77%)
Stage II	5 (19.23%)	16 (30.77%)
Stage III	10 (38.46%)	28 (53.84%)
Stage IV	9 (34.61%)	5 (9.62%)

**Table 2:** Values of AP and RI thickness are expressed as mean ± SD. Recurrence of effusion within the LHBT sheath is expressed as a percentage (%). AP: axillary pouch; RI: rotator interval; LHBT: long head of the biceps tendon.

Total (*n* = 78)	CT with AC (*n* = 26)	CT without AC (*n* = 52)	*p*-Value
AP thickness, mm	3.96 ± 1.37	2.08 ± 0.40	
RI thickness, mm	2.54 ± 0.38	1.81 ± 0.41	<0.00001
LHBT effusion, %	84.61	1579	<0.00001

## Data Availability

The raw data supporting the conclusions of this article will be made available by the authors on request.
